# Managing Ventricular Tachyarrhythmias In The Developing World: Insights From Recent ICD Trials

**Published:** 2001-10-01

**Authors:** KK Talwar, N Naik

Since the introduction of the implantable cardioverter defibrillator (ICD) by Michel Mirowski in the 1980's, these devices have become an important tenet in management of ventricular arrhythmias [1]. Numerous observational and randomized trials have demonstrated the superiority of this device over antiarrhythmic drugs in treating ventricular arrhythmias. The clinical implications of these trials are tremendous as sudden cardiac death (SCD) maybe the presenting symptom in these arrhythmias. It is estimated that in the United States alone around 350,000 deaths occur each year due to SCD alone [2]. As resuscitation is seldom successful in the community, methods to identify and appropriately treat such patients alone can prevent these events. However, the public health impact of this approach is tremendous, as the ICD is extremely expensive and is out of reach for many individuals. Moreover, not all patients with structural heart disease and ventricular arrhythmias have a malignant outcome. Thus efforts to appropriately stratify patients according to their risk profile and accordingly advocate ICD implant has important fiscal implications for the state with meager health resources, especially in those patients in whom survival benefit with the ICD is modest. 

Three major secondary prevention and two primary prevention trials have shown a survival benefit with the ICD [3-5]  The AVID trial was the largest of these and enrolled 1016 patients at high risk for VT/VF recurrences including SCD survivors, patients with syncopal VT and patients with severe symptoms during VT (chest pain, presyncope or hypotension) and left ventricular dysfunction (ejection fraction <0.40) [3].  These patients were followed for a mean period of 18 months. This trial was essentially a comparison between ICD and amiodarone in therapy of malignant ventricular arrhythmias as the 97% of patients in the antiarrhythmic arm in were on amiodarone. The trial was prematurely terminated when it became apparent that patients in the ICD arm had a significant survival advantage. There were 122 deaths in the drug arm and only 80 deaths in the ICD arm (number of arrhythmic deaths prevented by the ICD have not been specified by AVID investigators). The absolute risk reduction in mortality by the ICD was 11% at 3 years (relative reduction of 31% at 3 years). The use of the ICD was associated with a modest survival benefit of only 2.7 months at 3 years of follow-up.

The CIDS trial was another large randomized multicenter trial comparing ICD with amiodarone4. In this trial 659 patients were followed-up for a mean period of 3 years. There was a non-significant reduction in the risk of death in ICD arm from 10.2% per year to 8.3% per year (19.7% relative risk reduction, p = 0.142). The risk of arrhythmic death was also reduced (although non-significantly) from 4.5% to 3% (32.8% relative risk reduction, p = 0.094). On a follow-up 6 years life expectancy was 4.58 years in the ICD arm and 4.35 years in amiodarone arm. Cost-effective analysis performed on the first 430 patients in this trial showed an incremental cost-effectiveness of Canadian dollars 213, 543 per year of life gained [6].

The CASH trial was the smallest of the randomized, multicenter secondary prevention trials. Patients resuscitated from cardiac arrest secondary to documented VT/ VF unrelated to myocardial infarction were randomized to ICD, amiodarone, metoprolol or propafenone. The mean follow-up was for 51 ± 34 months. The propafenone arm of the trial was discontinued when an interim analysis demonstrated a 61% higher all-cause mortality in propafenone treated patients. The crude death rate in the ICD arm was 36.4% and 44.4% in the drug arm. The crude death rates in both amiodarone and metoprolol arms were similar. The ICD was associated with a better survival at 5 years although the difference was statistically non-significant (23% reduction in all-cause mortality, p = 0.081, hazard ratio 0.76). This probably results from the recruitment of a relatively healthy population compared from the AVID database (as reflected in the higher mean left ventricular ejection fraction).

The failure of these trials to unequivocally demonstrate a clear survival benefit with the ICD shows that not all patients are at a high risk of arrhythmic death. For instance, 15% of the CIDS population included patients who had unmonitored syncope who had a sustained monomorphic tachycardia on programmed ventricular stimulation. As this group is at a lower risk for recurrent arrhythmic events than those who have had documented ventricular arrhythmias, this heterogeneity in trial population with different risk profiles highlighted that an across the board recommendation of an ICD may not be necessary in all patients with ventricular arrhythmias. Similarly, the CIDS investigators also enrolled patients who had a more malignant VT in the electrophysiology (EP) lab even if their clinical VT was a stable, hemodynamically tolerated arrhythmia. In a post-hoc analysis of predictors of mortality in patients on amiodarone, the CIDS investigators demonstrated that only older age, poor left ventricular function and NYHA functional class were associated with a poorer outcome [7]. Significantly, clinical variables such as syncope during VT, presentation with cardiac arrest/VF and presence of unstable ventricular arrhythmias at EP were not predictive of increased arrhythmic death. These findings are significant as these clinical variables are generally believed to portend a poor prognosis. Using a risk score that included three variables (age more than 70 years, left ventricular ejection fraction = 35% and NYHA class III or IV) the CIDS population could be divided into three groups - 25% of the trial population had no risk factors, 51% had one risk factor and 24% had two or more risk factors. Patients with two or more of these risk factors were found to have a poor prognosis. Thus, according to this data, three-fourths of patients treated with amiodarone alone would have done as well as those on ICD.

Left ventricular function is an important determinant of long-term outcome. The AVID investigators demonstrated that almost the entire survival benefit with the ICD was in those with an ejection fraction = 35%. In patients with an ejection fraction > 35%, the ICD did not afford any survival benefit [8]. Similar observations were made by these investigators when they applied the risk score from the CIDS trial to the AVID population [9]. Post-hoc analysis from the MADIT trial were also concordant with these results - there was no additional benefit with the ICD over amiodarone in patients with ejection fractions > 26% [10].

Surprisingly, another sub-study from the AVID trial reported that patients whose index arrhythmia was VF did not benefit with the ICD if they had undergone prior revascularization, had ejection fraction more than 27% and did not have evidence of atherosclerosis in the cerebrovascular bed [11].The mean survival over a follow-up of 3 years in this group was similar in both the ICD and amiodarone arms (survival difference between the two groups was 0.03 ± 0.12 years). These results appear paradoxical as VF is a lethal arrhythmia and thus patients with this arrhythmia are expected to have poorer outcomes. Anti-ischemic and anti-adrenergic effects of amiodarone along with revascularization have been hypothesized as the physiological mechanisms resulting in these outcomes. However, these results need to be replicated in a randomized trial as post-hoc analysis can skew results due to various biases outside the control of the investigator.

Some non-randomized trials have also lent support to the notion that not all patients with ventricular arrhythmias and coronary artery disease have a poor outcome. Sarter et al demonstrated that in 124 patients who had prior myocardial infarction and had hemodynamically stable VT, sudden death rate was 2.4% per year in patients who received EP-guided therapy (including arrhythmia surgery) [12]. Brugada et al also showed that only 3/140 (2.1%) of patients with hemodynamically stable ischemic VT died of sudden death over a period of 26 months [13].

Results from some other randomized trials, however seem to favor ICD even in low-risk patients. The Electrophysiologic study versus Electrocardiographic Monitoring (ESVEM) trial demonstrated that a stable VT during the index episode did not necessarily mean a similar arrhythmia at recurrence [14]. Bocker et al showed that the VT at recurrence was a faster, hemodynamically unstable rhythm in 22% of those who had a stable VT at initial presentation [15]. Indeed, the AVID registry data demonstrated a non-significant increase in mortality in patients with stable as opposed to unstable VT (33.6% versus 27.6% at 3 years; relative risk 1.22, p = 0.07) [16].

These limitations from randomized trials have shown that not all patients are exposed to the same degree of risk of arrhythmia recurrence after their index episode. Certain groups of patients may do as well on anti-arrhythmic drugs. These include patients with relatively preserved ventricular function and stable ventricular tachycardias. Those patients whose index episode was VF and have been adequately revascularized and have no evidence of residual ischemia may also do well on antiarrhythmic drugs. The role of radiofrequency ablation in managing ventricular arrhythmias in coronary artery disease is evolving and is increasingly being used in many centers. Although it is still a challenging procedure, the availability of newer mapping techniques including non-fluoroscopic mapping systems and the Ensite system have made mapping of ischemic VT more plausible. A combination of drugs and radiofrequency ablation maybe effective antiarrhythmic therapy for some patients. Beta-blockers remain underutilized despite evidence that they reduce arrhythmia recurrence. Lipid-lowering agents have also been shown to reduce arrhythmia recurrence in one study. Optimized antiarrhythmic drug therapy using this combination of agents needs to be compared with the ICD in their efficacy to prevent arrhythmia recurrence. The issue of ICD unit cost too needs be given a serious thought. Relatively simpler versions of the ICD that have limited shocking abilities need to be devised so that it can be available to a larger patient base - as termed by Zipes, settling for a Volkswagen instead of a Rolls Royce [17].

It should be borne in mind that the vast majority of patients who experience a sudden cardiac death episode succumb to it in the index episode itself (only 5% of such patients survive). Thus if any substantial improvements are to be made in altering the natural history of these group patients, bystander resuscitative measures have to be markedly improved. This will involve not only education about cardiopulmonary resuscitation to the layman but also availability of automatic external defibrillators to aid in resuscitation of out-of-hospital cardiac arrest [18]. These devices analyze the cardiac rhythm of the patient and if appropriate deliver an external shock to convert VT/VF. The device is easy to use and contains audio-visual aids to guide the user throughout the process. An amalgamation of the above measures should streamline the approach of the physician and better help him to tackle this problem of modern cardiology.
